# Supplementary material for: Mena regulates nesprin-2 to control actin–nuclear lamina associations, trans-nuclear membrane signalling and gene expression
Source: Nat Commun. 2023 Mar 23;14:1602. doi: 10.1038/s41467-023-37021-x (PMC10036544; doi:10.1038/s41467-023-37021-x)
Supplement: Supplementary file 1 — Supplementary Information [file 41467_2023_37021_MOESM1_ESM.pdf]

Mena regulates nesprin-2 to control actin–nuclear lamina associations, trans-nuclear membrane signalling and gene expression

Frederic Li Mow Chee et al.

Supplementary Information

Supplementary Figure 1

Supplementary Figure 2

Supplementary Figure 3

Supplementary Figure 4

Supplementary Table 1

Supplementary Table 2

Correspondence should be addressed to Adam Byron (e-mail: adam.byron@manchester.ac.uk)

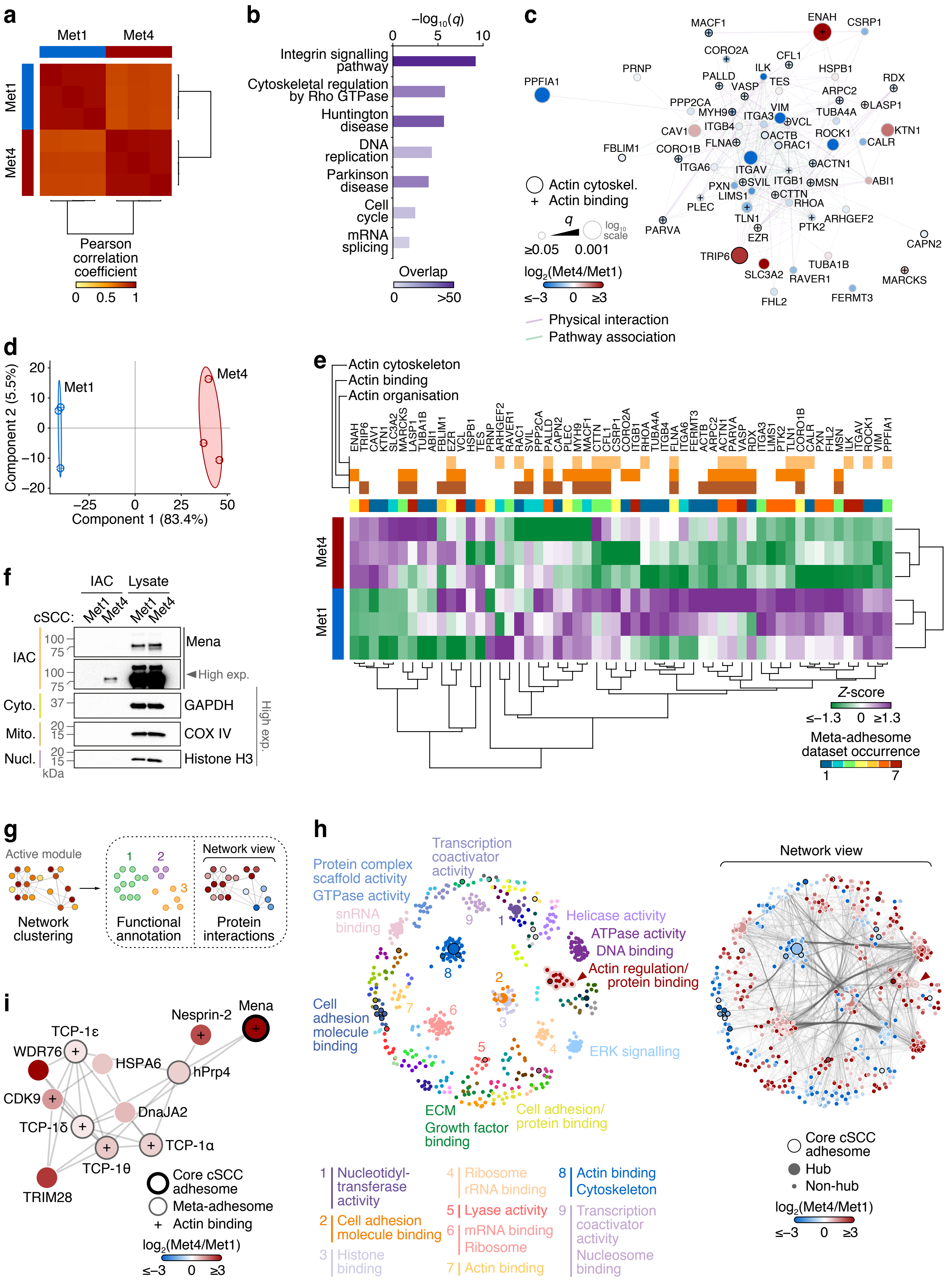

Supplementary Fig. 1 | See next page for caption.

Supplementary Fig. 1 | Proteomic analysis of patient-derived cSCC IACs. a Sample correlation analysis of mass spectrometric analyses of Met1 and Met4 IACs ($n = 3$ independent biological replicates). Pearson correlation coefficients for all pairwise sample comparisons were subjected to hierarchical clustering. **b** Over-representation analysis of Panther pathways in the cSCC IAC subproteome. Purple shading intensity indicates size of subproteome overlap with respective gene sets ($q < 0.05$, hypergeometric test with Benjamini–Hochberg correction). **c** Functional association network of the core cSCC adhesome. Proteins (nodes) are coloured according to protein enrichment in Met1 or Met4 IACs and annotated with gene names for clarity. Node size represents significance of differential enrichment between Met1 and Met4 IACs (two-sided t-test with Benjamini–Hochberg correction; $n = 3$ independent biological replicates). Black node borders indicate actin-cytoskeletal proteins; actin-binding proteins are indicated by a plus sign. **d** Principal component analysis of Met1 and Met4 IACs. **e** Hierarchical cluster analysis of the core cSCC adhesome. Data are as for Fig. 1e with additional annotation. Frequency of occurrence in meta-adhesome datasets is indicated, and proteins are annotated with gene names for clarity. **f** Isolation of Mena in Met4 IACs as determined by western blotting. GAPDH (cyto., cytoplasmic), COX IV (mito., mitochondrial) and histone H3 (nucl., nuclear) were probed as non-adhesion control proteins. Images are representative of three independent experiments. High exp., high exposure of blot. **g** Overview of IAC active module analysis. Dashed box contains graphical representation of the graph-based visualisations shown in (**h**) and Fig. 1h. Protein clusters were annotated with over-represented functional categories (numbered colours; left) and were displayed with protein quantification and reported physical protein interactions (network view; right). **h** Maximal-scoring active module of the cSCC IAC interactome. The network was derived as for that in Fig. 1h, but it was partitioned using the constant Potts model, resolved for maximum Surprise quality function. Proteins (nodes) are coloured according to assigned cluster (left panel). Molecular functions enriched in protein clusters were determined by Gene Ontology over-representation analysis (the two most significant functional categories are labelled). For protein clusters with fewer than two significant functional categories, clusters were annotated manually with a single representative term. Network view (right panel) shows corresponding protein interactions (edge densities) in the partitioned network; nodes are coloured according to protein enrichment in Met1 or Met4 IACs. The actin regulation cluster detailed in (**i**) is indicated with a red arrowhead. Large nodes indicate kinless and connector hubs; black node borders indicate core adhesome proteins. **i** Subnetwork analysis of the actin regulation cluster identified by active module partitioning in (**h**). The weighted subnetwork was clustered using a force-directed algorithm. Nodes are coloured according to protein enrichment in Met1 or Met4 IACs.

---

a

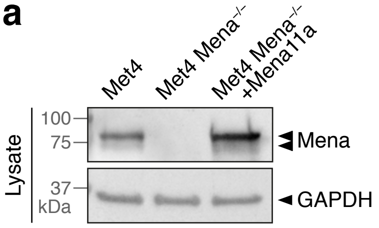

b

```
Met4      GGATCAACAATAGAAACAGAACAAAAAGAGGACAAAGGTGAAGATTCAGAGCCTGTAACT  355
Pan-Mena  GGATCAACAATAGAAACAGAACAAAAAGAGGACAAAGGTGAAGATTCAGAGCCTGTAACT  1500
Mena11a   GGATCAACAATAGAAACAGAACAAAAAGAGGACAAAGGTGAAGATTCAGAGCCTGTAACT  1500
          ************************************************************

Met4      TCTAAGGCCTCTTCAACAAGTACACCTGAACCAACAAGAAAACCTTGGGAAAGAACAAAT  415
Pan-Mena  TCTAAGGCCTCTTCAACAAGTACACCTGAACCAACAAGAAAACCTTGGGAAAGAACAAAT  1560
Mena11a   TCTAAGGCCTCTTCAACAAGTACACCTGAACCAACAAGAAAACCTTGGGAAAGAACAAAT  1560
          ************************************************************

Met4      ACAATGAATGGCAGCAAGTCACCTGTTATCTCCAGACGGGATTCTCCAAGGAAAAATCAG  475
Pan-Mena  ACAATGAATGGCAGCAAGTCACCTGTTATCTCCAGA------------------------  1596
Mena11a   ACAATGAATGGCAGCAAGTCACCTGTTATCTCCAGACGGGATTCTCCAAGGAAAAATCAG  1620
          ************************************

Met4      ATTGTTTTTGACAACAGGTCCTATGATTCATTACACAGACCAAAATCCACACCCTTATCA  535
Pan-Mena  ---------------------------------------CCAAAATCCACACCCTTATCA  1617
Mena11a   ATTGTTTTTGACAACAGGTCCTATGATTCATTACACAGACCAAAATCCACACCCTTATCA  1680
                                                 *********************

Met4      CAGCCCAGTGCCAATGGAGTCCAGACGGAAGGACTTGACTATGACAGGCTGAAGCAGGAC  595
Pan-Mena  CAGCCCAGTGCCAATGGAGTCCAGACGGAAGGACTTGACTATGACAGGCTGAAGCAGGAC  1677
Mena11a   CAGCCCAGTGCCAATGGAGTCCAGACGGAAGGACTTGACTATGACAGGCTGAAGCAGGAC  1740
          ************************************************************

Met4      ATTTTAGATGAAATGAGAAAAGAATTAACAAAGCTAAAAGAAGAGCTCATTGATGCAATC  655
Pan-Mena  ATTTTAGATGAAATGAGAAAAGAATTAACAAAGCTAAAAGAAGAGCTCATTGATGCAATC  1737
Mena11a   ATTTTAGATGAAATGAGAAAAGAATTAACAAAGCTAAAAGAAGAGCTCATTGATGCAATC  1800
          ************************************************************
```

Supplementary Fig. 2 | Analysis of Mena expression in Met4 cSCC cells. a Western blot analysis of endogenous Mena in parental Met4 cells, absence of Mena in Mena-depleted Met4 cells (Met4 Mena$^{-/-}$) and re-expression of Mena11a in Met4 Mena$^{-/-}$ cells (Met4 Mena$^{-/-}$ +Mena11a). Images are representative of three independent experiments. **b** Nucleotide sequence alignment of endogenous Mena transcript sequenced from Met4 cells. Mena sequence compared to pan-Mena (GenBank accession KM214203.1) and Mena11a (GenBank accession KM214204.1) coding sequences. Grey boxes indicate the additional Mena11a exon coding sequence. Asterisks indicate exact residue conservation.

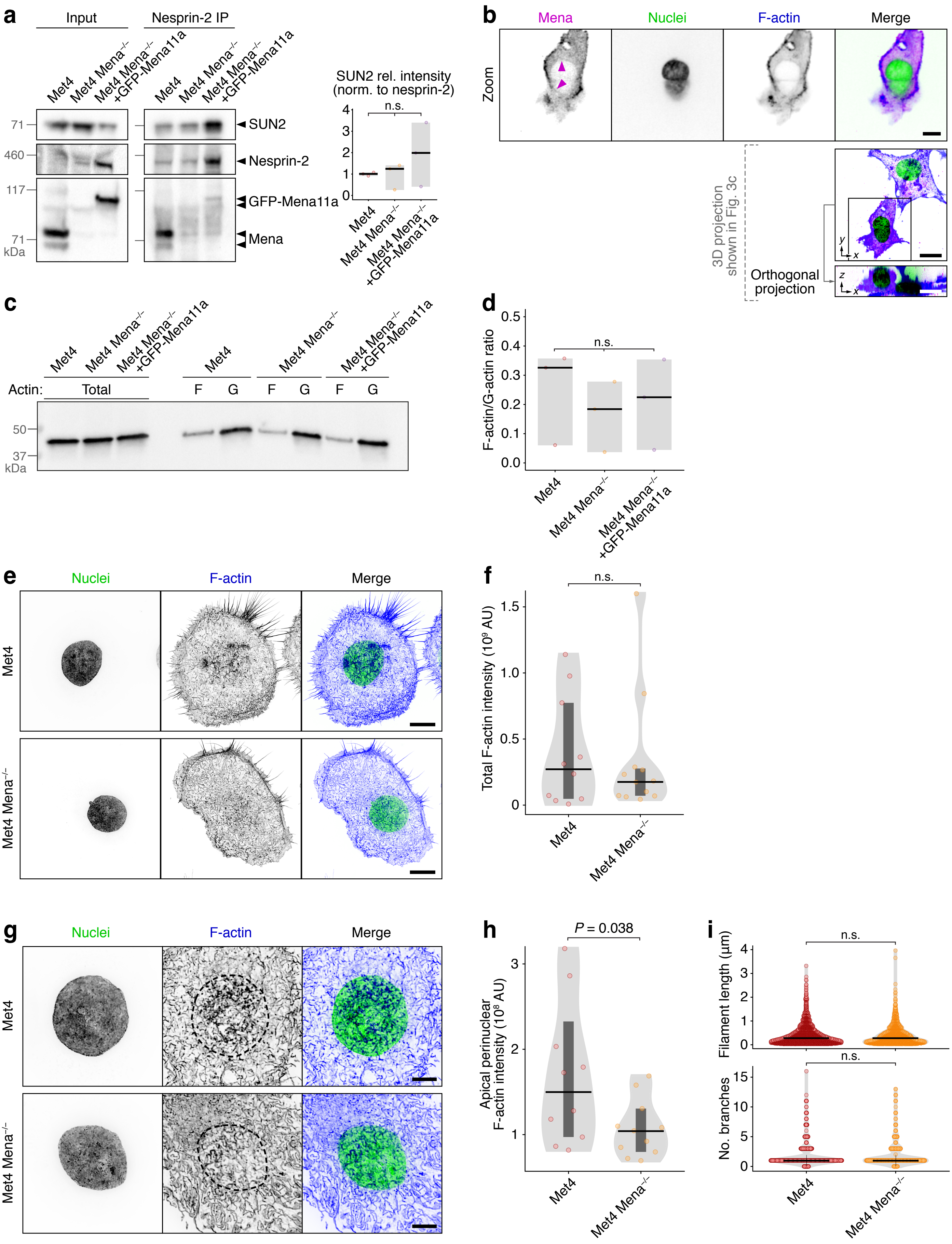

Supplementary Fig. 3 | See next page for caption.

Supplementary Fig. 3 | Analysis of actin in the presence and absence of Mena. a IP analysis of nesprin-2 protein complexes in Met4 cells, Mena-depleted Met4 cells (Met4 Mena$^{-/-}$) and Met4 Mena$^{-/-}$ cells with GFP-Mena11a re-expressed (Met4 Mena$^{-/-}$ +GFP-Mena11a). SUN2 was detected by western blotting; densitometric intensities were normalised (norm.) to nesprin-2 and expressed relative (rel.) to Met4 cells (right panel). Black bar, median; light grey box, range. Statistical analysis, one-way ANOVA (n = 3 independent experiments). **b** Spinning-disk confocal imaging of Met4 cells in 3D collagen matrix. 3D projections are from Fig. 3c; zoom images are a single central z-slice. Magenta arrowheads indicate localisation of Mena at the nuclear periphery. Scale bar, 20 µm; zoom (inset) scale bar, 10 µm. **c** Western blot analysis of polymeric F-actin and monomeric G-actin fractions isolated from Met4, Met4 Mena$^{-/-}$ and Met4 Mena$^{-/-}$ +GFP-Mena11a cells. **d** Ratios of F-actin to G-actin (see **c**). Black bar, median; light grey box, range. Statistical analysis, one-way ANOVA (n = 3 independent experiments). **e** Spinning-disk confocal imaging of Met4 cells and Met4 Mena$^{-/-}$ cells. Scale bar, 10 µm. **f** Quantification of whole-cell F-actin mean fluorescence intensity (see **e**). Black bar, median; dark grey box, 95% confidence interval; light grey silhouette, probability density. Statistical analysis, two-sided Mann–Whitney test (n = 10 and 12 cells for Met4 and Met4 Mena$^{-/-}$ cells, respectively, from n = 3 independent experiments). **g** Optical pixel reassignment imaging of apical regions of Met4 cells and Met4 Mena$^{-/-}$ cells. Nuclear perimeters delimited the regions of interest (dashed black borders). Scale bar, 5 µm. For (**b**), (**e**) and (**g**), nuclei were detected using DAPI; F-actin was detected using phalloidin. Inverted lookup tables were applied. Images are representative of three independent experiments (two for **b**). **h, i** Quantification of apical perinuclear F-actin mean fluorescence intensity (**h**) and organisation (**i**) (see **g**). Black bar, median; dark grey box, 95% confidence interval; light grey silhouette, probability density. Statistical analysis, two-sided Student's t-test (n = 10 and 11 cells for Met4 and Met4 Mena$^{-/-}$ cells, respectively, from n = 3 independent experiments) for (**h**), two-sided Mann–Whitney tests (n = 2,218 and 1,575 actin filaments (length quantification) or n = 939 and 637 actin filaments (branch quantification) for Met4 and Met4 Mena$^{-/-}$ cells, respectively, from n = 3 independent experiments) for (**i**). n.s., not significant.

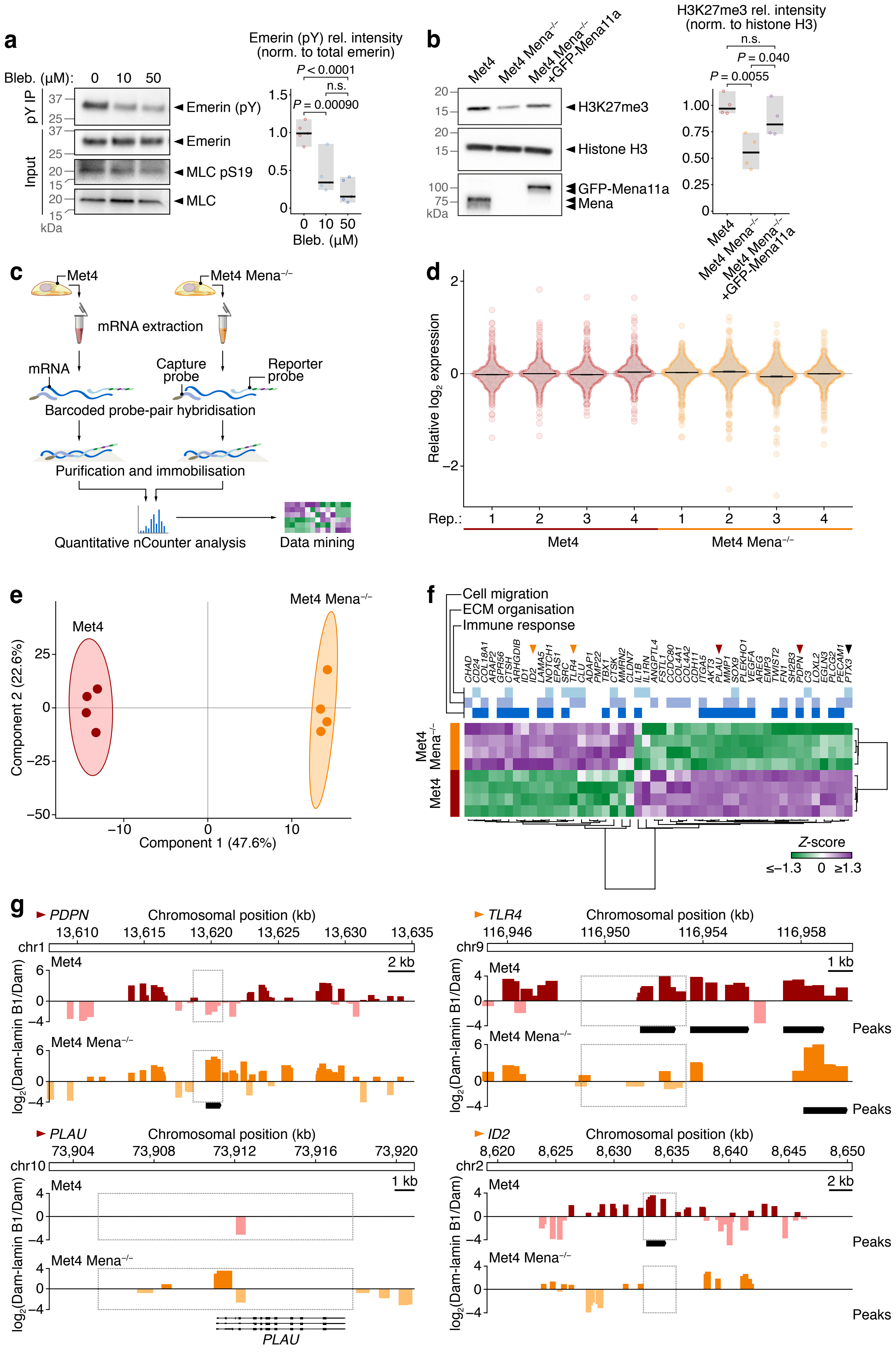

Supplementary Fig. 4 | See next page for caption.

Supplementary Fig. 4 | Regulation of cSCC gene expression by Mena. a Emerin tyrosine phosphorylation after treatment of cells with blebbistatin. Met4 cells were treated with 0 (vehicle control), 10 or 50 μM blebbistatin (bleb.) for 2 h. Emerin tyrosine phosphorylation was detected by phosphotyrosine (pY) IP followed by western blotting for emerin; densitometric intensities were normalised (norm.) to total emerin (lysate) and expressed relative (rel.) to vehicle control (right panel). Black bar, median; light grey box, range. Statistical analysis, one-way ANOVA with Tukey's correction ($n = 5$ independent experiments). MLC, myosin light chain. **b** Western blot analysis of H3K27me3 in Met4, Met4 Mena$^{-/-}$ and Met4 Mena$^{-/-}$ +GFP-Mena11a cell lysates. Densitometric intensities were normalised to histone H3 and expressed relative to Met4 cells (right panel). Black bar, median; light grey box, range. Statistical analysis, one-way ANOVA with Tukey's correction ($n = 4$ independent experiments). **c** Workflow for multiplexed digital quantification of the expression of 770 genes associated with cancer progression. Dark blue strand, messenger RNA (mRNA). **d** Normalised cancer progression gene expression data across independent biological replicate (rep.) analyses for Met4 and Met4 Mena$^{-/-}$ cells. Black bar, median; dark grey box, 95% confidence interval; light grey silhouette, probability density. Most 95% confidence intervals are smaller than the weight of the corresponding median line (black bar) and so are not visible. **e** Principal component analysis of cancer progression gene expression for Met4 and Met4 Mena$^{-/-}$ cells. **f** Hierarchical cluster analysis of cancer progression genes significantly differentially regulated between Met4 and Met4 Mena$^{-/-}$ cells ($q < 0.05$, two-sided t-test with Benjamini–Hochberg correction; $n = 4$ independent biological replicates). Data are as for Fig. 4b with additional annotation. The most downregulated gene upon Mena depletion, *PTX3*, is indicated with a black arrowhead; the next two most downregulated genes in Mena-deficient cells, *PDPN* and *PLAU*, are indicated with red arrowheads; the two most upregulated genes in Mena-deficient cells, *TLR4* and *ID2*, are indicated with orange arrowheads. **g** Nuclear lamina association profiles identified by lamin B1 DamID sequencing (DamID-seq). Lamin B1 DamID-seq tracks were generated from Met4 (red, top profile) and Met4 Mena$^{-/-}$ (orange, bottom profile) cells, and regions spanning putative enhancer regions for the most dysregulated genes identified by multiplexed gene expression analysis (arrowheads in **f**) were selected. Tracks spanning enhancer regions associated with *PDPN* (27-kb region of chromosome 1; top left panel), *PLAU* (18-kb region of chromosome 10; bottom left panel), *TLR4* (15-kb region of chromosome 9; top right panel) and *ID2* (31-kb region of chromosome 2; bottom right panel) are shown (human reference genome GRCh38). Tracks spanning the enhancer region associated with *PTX3* are shown in Fig. 4e. Enhancer regions associated with *PDPN* (GeneHancer identifier GH01J013618), *PLAU* (GeneHancer identifier GH10J073905), *TLR4* (GeneHancer identifier GH09J116950) and *ID2* (GeneHancer identifier GH02J008632) are indicated with dotted boxes. Significantly called DamID peaks are indicated with black bars (FDR < 5%; $n = 2$ independent experiments). Scale bar, 2 kb (*PDPN* and *ID2*) or 1 kb (*PLAU* and *TLR4*). n.s., not significant.

Supplementary Table 1 | Oligonucleotides used in this study

Purpose	Name	Sequence (5′–3′)
CRISPR/Cas9	*ENAH* exon 2 guide, forward	TTTCTTGGCTTTATATATCTTGTGGAAAGGACGAAACACCGGAGATGCTAGACAGGTGTA
CRISPR/Cas9	*ENAH* exon 2 guide, reverse	GACTAGCCTTATTTTAACTTGCTATTTCTAGCTCTAAAACTACACCTGTCTAGCATCTCC
Sequencing	*ENAH*, forward	ATGAGTGAACAGAGTATCTG
Sequencing	*ENAH*, reverse	CTATGCAGTATTTGACTTGC
Sequencing	M13, forward	TGTAAAACGACGGCCAGT
Sequencing	M13, reverse	CAGGAAACAGCTATGAC
DamID	AdR_PCR	GGTCGCGGCCGAGGATC
DamID	Ad1_noMX	AATGATACGGCGACCACCGAGATCTACACTCGTCGGCAGCGTCAGATGTG
DamID	Ad2.3	CAAGCAGAAGACGGCATACGAGATTTCTGCCTGTCTCGTGGGCTCGGAGATGT
DamID	Ad2.4	CAAGCAGAAGACGGCATACGAGATGCTCAGGAGTCTCGTGGGCTCGGAGATGT
DamID	Ad2.5	CAAGCAGAAGACGGCATACGAGATAGGAGTCCGTCTCGTGGGCTCGGAGATGT
DamID	Ad2.6	CAAGCAGAAGACGGCATACGAGATCATGCCTAGTCTCGTGGGCTCGGAGATGT
DamID	Ad2.7	CAAGCAGAAGACGGCATACGAGATGTAGAGAGGTCTCGTGGGCTCGGAGATGT
DamID	Ad2.8	CAAGCAGAAGACGGCATACGAGATCCTCTCTGGTCTCGTGGGCTCGGAGATGT
qPCR	*PTX3* RT-PCR, forward	CGTCTCTCCAGCAATGCATC
qPCR	*PTX3* RT-PCR, reverse	AAGAGCTTGTCCCATTCCGA
qPCR	*GAPDH* RT-PCR, forward	GGACCTGACCTGCCGTCTAG
qPCR	*GAPDH* RT-PCR, reverse	TGGTGCTCAGTGTAGCCCAG
qPCR	*PTX3* DamID (TF2), forward	TACAAGGCGGGAAAATTGGC
qPCR	*PTX3* DamID (TF2), reverse	TATAACGGGCCAGTGGACTC

Supplementary Table 2 | STED microscopy laser settings

			Wavelength (nm)			
Dye	Brand	Supplier	Excitation	Detection	Depletion	Time gating (ns)
CF680R	Biotium	Cambridge Bioscience	670	680–750	775	1.5–8.0
STAR RED	Abberior	Sigma-Aldrich	635	645–670	775	0.3–8.0
STAR 580	Abberior	Sigma-Aldrich	590	600–640	775	0.3–8.0
STAR 488	Abberior	Sigma-Aldrich	490	500–580	592	0.5–8.0
