# Supplementary material for: Mena regulates nesprin-2 to control actin–nuclear lamina associations, trans-nuclear membrane signalling and gene expression
Source: Nat Commun. 2023 Mar 23;14:1602. doi: 10.1038/s41467-023-37021-x (PMC10036544; doi:10.1038/s41467-023-37021-x)
Supplement: Supplementary file 2 — Reporting Summary [file 41467_2023_37021_MOESM2_ESM.pdf]

nature portfolio

Corresponding author(s): Adam Byron

Last updated by author(s): Feb 8, 2023

Reporting Summary

Nature Portfolio wishes to improve the reproducibility of the work that we publish. This form provides structure for consistency and transparency in reporting. For further information on Nature Portfolio policies, see our Editorial Policies and the Editorial Policy Checklist.

Statistics

For all statistical analyses, confirm that the following items are present in the figure legend, table legend, main text, or Methods section.

n/a	Confirmed	
☐	☒	The exact sample size (n) for each experimental group/condition, given as a discrete number and unit of measurement
☐	☒	A statement on whether measurements were taken from distinct samples or whether the same sample was measured repeatedly
☐	☒	The statistical test(s) used AND whether they are one- or two-sided<br>*Only common tests should be described solely by name; describe more complex techniques in the Methods section.*
☒	☐	A description of all covariates tested
☐	☒	A description of any assumptions or corrections, such as tests of normality and adjustment for multiple comparisons
☐	☒	A full description of the statistical parameters including central tendency (e.g. means) or other basic estimates (e.g. regression coefficient) AND variation (e.g. standard deviation) or associated estimates of uncertainty (e.g. confidence intervals)
☐	☒	For null hypothesis testing, the test statistic (e.g. F, t, r) with confidence intervals, effect sizes, degrees of freedom and P value noted<br>*Give P values as exact values whenever suitable.*
☒	☐	For Bayesian analysis, information on the choice of priors and Markov chain Monte Carlo settings
☒	☐	For hierarchical and complex designs, identification of the appropriate level for tests and full reporting of outcomes
☐	☒	Estimates of effect sizes (e.g. Cohen's d, Pearson's r), indicating how they were calculated

Our web collection on statistics for biologists contains articles on many of the points above.

Software and code

Policy information about availability of computer code

Data collection	Image Lab (version 5.2.1; Bio-Rad).
Data analysis	Image Lab (version 5.2.1; Bio-Rad); NIS-Elements Advanced Research (versions 4.6 and 5.21.03; Nikon Instruments); Imaris (versions 9.2.0 and 9.8.0; Oxford Instruments); Fiji (versions 1.52p and 1.53c); MaxQuant (version 1.5.3.17); Normalyzer (version 1.1.1); MICE (version 3.9.0); WebGestalt (version 2019); BioNet (version 1.46.0); Heinz (version 1.63); igraph package (version 1.2.5; https://igraph.org); louvain package (version 0.6.0; https://pypi.org/project/louvain); Cytoscape (version 3.6.1 and version 3.8.0); GeneMANIA (version 3.5.1); Cluster 3.0 (C Clustering Library, version 1.54); Java TreeView (version 1.1.5r2); nSolver Analysis (version 4.0; NanoString Technologies); TrimGalore (version 0.6.3); BBMap (version 38.11); Bowtie 2 (version 2.3.4.3); SAMtools (version 1.6); Integrated Genome Browser (version 9.1.4); find_peaks script (version 1.0.1; https://github.com/owenjm/find_peaks); limma (version 3.11); Excel (version 16.16.26; Microsoft); PlotsOfData (version 1.0.5).

For manuscripts utilizing custom algorithms or software that are central to the research but not yet described in published literature, software must be made available to editors and reviewers. We strongly encourage code deposition in a community repository (e.g. GitHub). See the Nature Portfolio guidelines for submitting code & software for further information.

Data

Policy information about availability of data

All manuscripts must include a data availability statement. This statement should provide the following information, where applicable:

- Accession codes, unique identifiers, or web links for publicly available datasets
- A description of any restrictions on data availability
- For clinical datasets or third party data, please ensure that the statement adheres to our policy

The MS-based proteomics data generated in this study have been deposited in ProteomeXchange via the PRIDE partner repository with dataset accession identifier PXD021492 [http://proteomecentral.proteomexchange.org/cgi/GetDataset?ID=PXD021492]. The DamID sequencing data generated in this study have been deposited in the Gene Expression Omnibus with GEO series accession identifier GSE159598 [https://www.ncbi.nlm.nih.gov/geo/query/acc.cgi?acc=GSE159598]. The protein sequence data used in this study are available in the UniProtKB database with release identifier 2015_09 [https://ftp.uniprot.org/pub/databases/uniprot/previous_releases/release-2015_09/]. The human reference genome data used in this study are available from the Genome Reference Consortium with release identifier GRCh38 [https://ftp.ncbi.nlm.nih.gov/genomes/all/GCA/000/001/405/GCA_000001405.15_GRCh38]. All other data supporting the findings of this study are available within the paper and its Supplementary Information and Source Data files. Requests for materials should be addressed to Adam Byron. Source data are provided with this paper.

Human research participants

Policy information about studies involving human research participants and Sex and Gender in Research.

Reporting on sex and gender	The cell lines, described previously (Proby et al. (2000) Exp. Dermatol. 9, 104–117 https://doi.org/10.1034/j.1600-0625.2000.009002104.x; Hassan et al. (2019) Int. J. Mol. Sci. 20, 3428 https://doi.org/10.3390/ijms20143428), were derived from a single male cutaneous squamous cell carcinoma (cSCC) patient.
Population characteristics	The patient was a 45-year-old male renal transplant cSCC patient that was receiving immunotherapy (azathioprine, prednisolone), as described previously (Proby et al. (2000) Exp. Dermatol. 9, 104–117 https://doi.org/10.1034/j.1600-0625.2000.009002104.x; Hassan et al. (2019) Int. J. Mol. Sci. 20, 3428 https://doi.org/10.3390/ijms20143428).
Recruitment	The patient was recruited via attendance at a dermatology clinic following clinical diagnosis of a cutaneous SCC lesion, and the patient provided written, informed consent, as described previously (Proby et al. (2000) Exp. Dermatol. 9, 104–117 https://doi.org/10.1034/j.1600-0625.2000.009002104.x; Hassan et al. (2019) Int. J. Mol. Sci. 20, 3428 https://doi.org/10.3390/ijms20143428).
Ethics oversight	Ethical approval (REC reference 08/S1401/69, 5 November 2008) was obtained from the East London and City Health Authority local ethics committee, and the study was conducted according to the Declaration of Helsinki Principles.

Note that full information on the approval of the study protocol must also be provided in the manuscript.

Field-specific reporting

Please select the one below that is the best fit for your research. If you are not sure, read the appropriate sections before making your selection.

☒ Life sciences ☐ Behavioural & social sciences ☐ Ecological, evolutionary & environmental sciences

For a reference copy of the document with all sections, see nature.com/documents/nr-reporting-summary-flat.pdf

Life sciences study design

All studies must disclose on these points even when the disclosure is negative.

Sample size	Sample size was not predetermined in this study. The number of independent experimental samples used for each experiment is indicated in the Methods section or corresponding figure legend. Sample size was determined empirically from previous experimental evidence with similar assays to be sufficient to detect minimal biologically relevant differences between experimental conditions at the 5% significance level (Canel et al. (2017) Cancer Res. 77, 5301–5312; Serrels et al. (2017) Sci. Signal. 10, eaan8355; Schoenherr et al. (2020) J. Biol. Chem. 295, 12045–12057; Patel et al. (2020) Sci. Rep. 10, 3902; Randles et al. (2020) Matrix Biol. 90, 61-78; Acebrón et al. (2020) EMBO J. 39, e104743; Griffith et al. (2021) Sci. Rep. 11, 229; Rabanal-Ruiz et al. (2021) J. Cell Biol. 220, e202004010; Byron et al. (2022) Nat. Commun. 13, 3053).
Data exclusions	For MS data analysis, proteins that were not quantified in at least two out of three independent biological replicates for at least one experimental condition were excluded from further analysis to minimise missing values, improve missing value imputation across the dataset and improve accuracy of quantification. For multiplexed gene expression analysis (nCounter), a minimum mean count threshold was set to 20 counts to remove features with very low signal. For DamID-qPCR, one independent biological replicate was excluded as a corresponding data point was an outlier by three orders of magnitude.
Replication	Experimental findings were replicated using multiple independent biological replicates as indicated in the Methods section or corresponding figure legend.

Randomization | The same number of cells were randomly apportioned to each sample.

Blinding | Experiments in this study were performed non-blinded to experimental group allocation as complicated experimental designs rendered blinding unfeasible or sample allocation concealment would hinder apportioning of correct cell types or applying distinct experimental treatments to samples. The risk of bias was assessed as low as the experiments were routinely performed by adequately trained personnel using well-established, standardised methods. For computational analyses, experimental group allocation was not provided to the algorithms.

Reporting for specific materials, systems and methods

We require information from authors about some types of materials, experimental systems and methods used in many studies. Here, indicate whether each material, system or method listed is relevant to your study. If you are not sure if a list item applies to your research, read the appropriate section before selecting a response.

Materials & experimental systems

n/a	Involved in the study
☐	☒ Antibodies
☐	☒ Eukaryotic cell lines
☒	☐ Palaeontology and archaeology
☒	☐ Animals and other organisms
☒	☐ Clinical data
☒	☐ Dual use research of concern

Methods

n/a	Involved in the study
☒	☐ ChIP-seq
☒	☐ Flow cytometry
☒	☐ MRI-based neuroimaging

Antibodies

Antibodies used | The following primary antibodies were used (all Cell Signaling Technology, diluted 1:1,000, unless otherwise stated): anti-αV integrin (clone EPR16800; Abcam, #ab179475), anti-actin (clone 13E5; #4970; and clone 8H10D10; #3700), anti-cytochrome c oxidase subunit 4 (clone 4D11-B3-E8; #11967), anti-emerin (clone D3B9G; #30853), anti-FAK (#3285), anti-FAK pY397 (#3283), anti-GAPDH (clone D16H11; #5174), anti-GFP (BioVision, #3999-100), anti-H3K27me3 (clone C36B11; #9733), anti-histone H3 (clone D1H2; #4499), anti-lamin A/C (#2032), anti-Mena (Atlas, #HPA028448; and clone A351F7D9; Merck Millipore, #MAB2635), anti-myosin light chain 2 (clone D18E2; #8505), anti-myosin light chain 2 pS19 (#3675), anti-nesprin-2 (Abcam, #ab217057; and Abcam, #ab204308), anti-phosphotyrosine (clone PY20; BD Transduction, #610000). The following secondary antibodies were used (all diluted 1:400 unless otherwise stated): anti-rabbit IgG conjugated to horseradish peroxidase (Cell Signaling Technology, #7074, diluted 1:10,000), anti-mouse IgG conjugated to horseradish peroxidase (Cell Signaling Technology, #7076, diluted 1:5,000), anti-rabbit IgG conjugated to Alexa Fluor 594 (Thermo Fisher Scientific, #A-11072), anti-mouse IgG conjugated to ATTO 647N (Rockland Immunochemicals, #610-156-121), anti-rabbit IgG conjugated to Abberior STAR RED (Abberior, #STRED-1002-500UG), anti-mouse IgG conjugated to Abberior STAR 580 (Abberior, #ST580-1001-500UG).

Validation | Detailed information on the antibodies used, including validation information and relevant references, is provided via the Resource Identification Portal [https://scicrunch.org/resources] (RRID antibody IDs provided below where available) and the respective manufacturer's website (all Cell Signaling Technology unless otherwise stated) as follows:
- anti-αV integrin (clone EPR16800; Abcam, #ab179475) [https://www.abcam.com/integrin-alpha-v-antibody-epr16800-ab179475.html], RRID:AB_2716738 [https://antibodyregistry.org/search.php?q=AB_2716738]
- anti-actin (clone 13E5; #4970) [https://www.cellsignal.com/products/primary-antibodies/b-actin-13e5-rabbit-mab/4970], RRID:AB_2223172 [https://antibodyregistry.org/search.php?q=AB_2223172]
- anti-actin (clone 8H10D10; #3700) [https://www.cellsignal.com/products/primary-antibodies/b-actin-8h10d10-mouse-mab/3700], RRID:AB_2242334 [https://antibodyregistry.org/search.php?q=AB_2242334]
- anti-cytochrome c oxidase subunit 4 (clone 4D11-B3-E8; #11967) [https://www.cellsignal.com/products/primary-antibodies/cox-iv-4d11-b3-e8-mouse-mab/11967], RRID:AB_2797784 [https://antibodyregistry.org/search.php?q=AB_2797784]
- anti-emerin (clone D3B9G; #30853) [https://www.cellsignal.com/products/primary-antibodies/emerin-d3b9g-xp-rabbit-mab/30853], RRID:AB_2798996 [https://antibodyregistry.org/search.php?q=AB_2798996]
- anti-FAK (#3285) [https://www.cellsignal.com/products/primary-antibodies/fak-antibody/3285], RRID:AB_2269034 [https://antibodyregistry.org/search.php?q=AB_2269034]
- anti-FAK pY397 (#3283) [https://www.cellsignal.com/products/primary-antibodies/phospho-fak-tyr397-antibody/3283], RRID:AB_2173659 [https://antibodyregistry.org/search.php?q=AB_2173659]
- anti-GAPDH (clone D16H11; #5174) [https://www.cellsignal.com/products/primary-antibodies/gapdh-d16h11-xp-rabbit-mab/5174], RRID:AB_10622025 [https://antibodyregistry.org/search.php?q=AB_10622025]
- anti-GFP (BioVision, #3999-100) [https://www.biovision.com/gfp-antibody-17047.html], RRID:AB_222261 [https://antibodyregistry.org/search.php?q=AB_222261]
- anti-H3K27me3 (clone C36B11; #9733) [https://www.cellsignal.com/products/primary-antibodies/tri-methyl-histone-h3-lys27-c36b11-rabbit-mab/9733], RRID:AB_2616029 [https://antibodyregistry.org/search.php?q=AB_2616029]
- anti-histone H3 (clone D1H2; #4499) [https://www.cellsignal.com/products/primary-antibodies/histone-h3-d1h2-xp-rabbit-mab/4499], RRID:AB_10544537 [https://antibodyregistry.org/search.php?q=AB_10544537]
- anti-lamin A/C (#2032) [https://www.cellsignal.com/products/primary-antibodies/lamin-a-c-antibody/2032], RRID:AB_2136278 [https://antibodyregistry.org/search.php?q=AB_2136278]
- anti-Mena (Atlas, #HPA028448) [https://www.atlasantibodies.com/products/antibodies/primary-antibodies/triple-a-polyclonals/enah-antibody-hpa028448/], RRID:AB_10600766 [https://antibodyregistry.org/search.php?q=AB_10600766]
- anti-Mena (clone A351F7D9; Merck Millipore, #MAB2635) [https://www.merckmillipore.com/GB/en/product/Anti-Mena-Antibody-clone-A351F7D9,MM_NF-MAB2635], RRID:AB_11214403 [https://antibodyregistry.org/search.php?q=AB_11214403]

- anti-myosin light chain 2 (clone D18E2; #8505) [https://www.cellsignal.com/products/primary-antibodies/myosin-light-chain-2-d18e2-rabbit-mab/8505], RRID:AB_2728760 [https://antibodyregistry.org/search.php?q=AB_2728760]
- anti-myosin light chain 2 pS19 (#3675) [https://www.cellsignal.com/products/primary-antibodies/phospho-myosin-light-chain-2-ser19-mouse-mab/3675], RRID:AB_2250969 [https://antibodyregistry.org/search.php?q=AB_2250969]
- anti-nesprin-2 (Abcam, #ab217057) [https://www.abcam.com/nesprin-2-antibody-ab217057.html]
- anti-nesprin-2 (Abcam, #ab204308) [https://www.abcam.com/nesprin-2-antibody-ab204308.html]
- anti-phosphotyrosine (clone PY20; BD Transduction, #610000) [https://www.bdbiosciences.com/en-gb/products/reagents/microscopy-imaging-reagents/immunofluorescence-reagents/purified-mouse-anti-phosphotyrosine.610000], RRID:AB_397423 [https://antibodyregistry.org/search.php?q=AB_397423]
- anti-rabbit IgG conjugated to horseradish peroxidase (Cell Signaling Technology, #7074, diluted 1:10,000) [https://www.cellsignal.com/products/secondary-antibodies/anti-rabbit-igg-hrp-linked-antibody/7074], RRID:AB_2099233 [https://antibodyregistry.org/search.php?q=AB_2099233]
- anti-mouse IgG conjugated to horseradish peroxidase (Cell Signaling Technology, #7076, diluted 1:5,000) [https://www.cellsignal.com/products/secondary-antibodies/anti-mouse-igg-hrp-linked-antibody/7076], RRID:AB_330924 [https://antibodyregistry.org/search.php?q=AB_330924]
- anti-rabbit IgG conjugated to Alexa Fluor 594 (Thermo Fisher Scientific, #A-11072) [https://www.thermofisher.com/antibody/product/Goat-anti-Rabbit-IgG-H-L-Cross-Adsorbed-Secondary-Antibody-Polyclonal/A-11072], RRID:AB_2534116 [https://antibodyregistry.org/search.php?q=AB_2534116]
- anti-mouse IgG conjugated to ATTO 647N (Rockland Immunochemicals, #610-156-121) [https://www.rockland.com/categories/secondary-antibodies/mouse-igg-hl-antibody-atto-647n-conjugated-pre-adsorbed-610-156-121/], RRID:AB_10894200 [https://antibodyregistry.org/search.php?q=AB_10894200]
- anti-rabbit IgG conjugated to Abberior STAR RED (Abberior, #STRED-1002-500UG) [https://abberior.shop/abberior-STAR-RED-goat-anti-rabbit-IgG-500-ll-1-mg-ml], RRID:AB_2833015 [https://antibodyregistry.org/search.php?q=AB_2833015]
- anti-mouse IgG conjugated to Abberior STAR 580 (Abberior, #ST580-1001-500UG) [https://abberior.shop/abberior-STAR-580-goat-anti-mouse-IgG-500-ll-1-mg-ml], RRID:AB_2923543 [https://antibodyregistry.org/search.php?q=AB_2923543].

Eukaryotic cell lines

Policy information about cell lines and Sex and Gender in Research

Cell line source(s)	Human cSCC Met1 and Met4 cells (malignant keratinocytes) were described previously (Hassan et al. (2019) Int. J. Mol. Sci. 20, 3428 https://doi.org/10.3390/ijms20143428): Met1 cells were derived from primary cSCC and Met4 cells from distant metastatic cSCC from the same immunosuppressed patient (see Proby et al. (2000) Exp. Dermatol. 9, 104–117 https://doi.org/10.1034/j.1600-0625.2000.009002104.x). Met1 subclone 1 and Met4 subclone 1 were established following cell culture and used for the experiments herein. HEK293T cells were a gift from Noor Gammoh (University of Edinburgh, Edinburgh UK).
Authentication	None of the cell lines used were authenticated.
Mycoplasma contamination	Cell lines were routinely tested for mycoplasma and used within three months of recovery from frozen.
Commonly misidentified lines (See ICLAC register)	None used.
