# Supplementary material for: Mena regulates nesprin-2 to control actin–nuclear lamina associations, trans-nuclear membrane signalling and gene expression
Source: Nat Commun. 2023 Mar 23;14:1602. doi: 10.1038/s41467-023-37021-x (PMC10036544; doi:10.1038/s41467-023-37021-x)
Supplement: Supplementary file 4 — Description of Additional Supplementary Files [file 41467_2023_37021_MOESM4_ESM.pdf]

File name: Supplementary Data 1
Description: MS-based proteomic analysis of patient-derived cSCC integrin adhesion complexes.

File name: Supplementary Data 2
Description: Core cSCC adhesion proteins quantified by LC-MS/MS.

File name: Supplementary Data 3
Description: Multiplexed gene expression analysis of detected cancer progression genes.

File name: Supplementary Data 4
Description: Differentially regulated cancer progression genes upon Mena depletion.
