# Supplementary material for: Mena regulates nesprin-2 to control actin–nuclear lamina associations, trans-nuclear membrane signalling and gene expression
Source: Nat Commun. 2023 Mar 23;14:1602. doi: 10.1038/s41467-023-37021-x (PMC10036544; doi:10.1038/s41467-023-37021-x)
Supplement: Supplementary file 9 — Source Data [file 41467_2023_37021_MOESM9_ESM.zip › LiMowChee_SourceData_Blots.pdf]

Fig. 1b

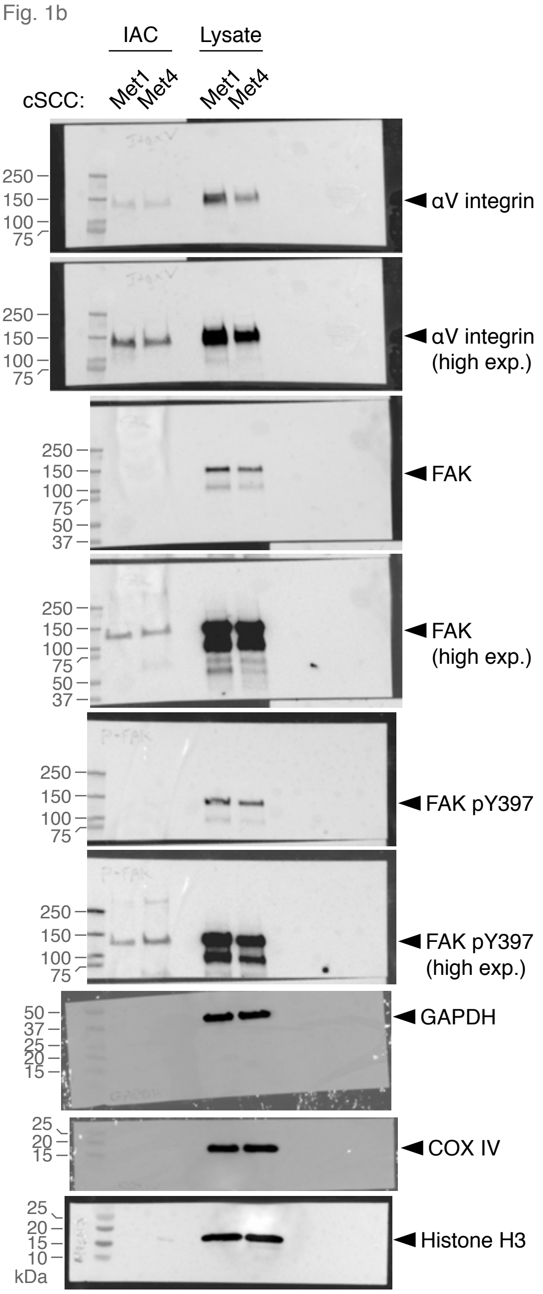

Fig. 2e

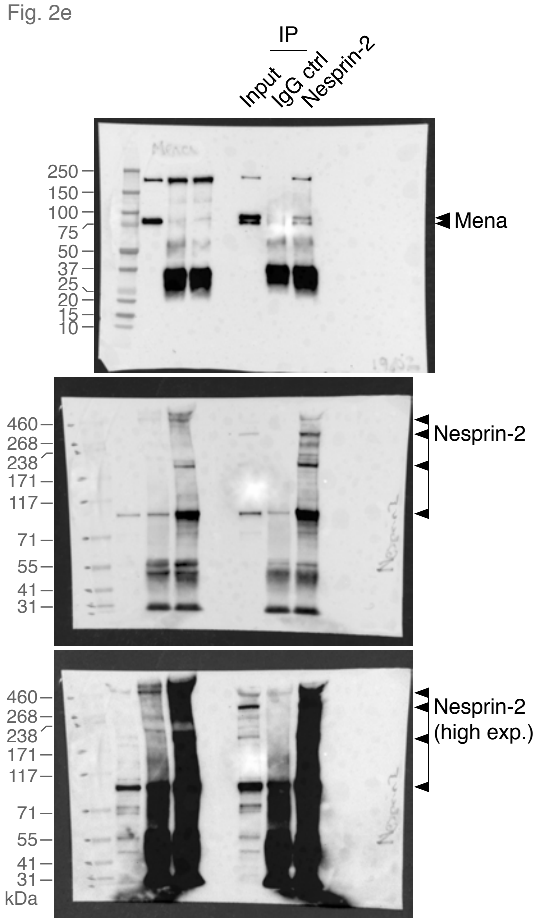

Source Data. Continued on next page.

Fig. 2g

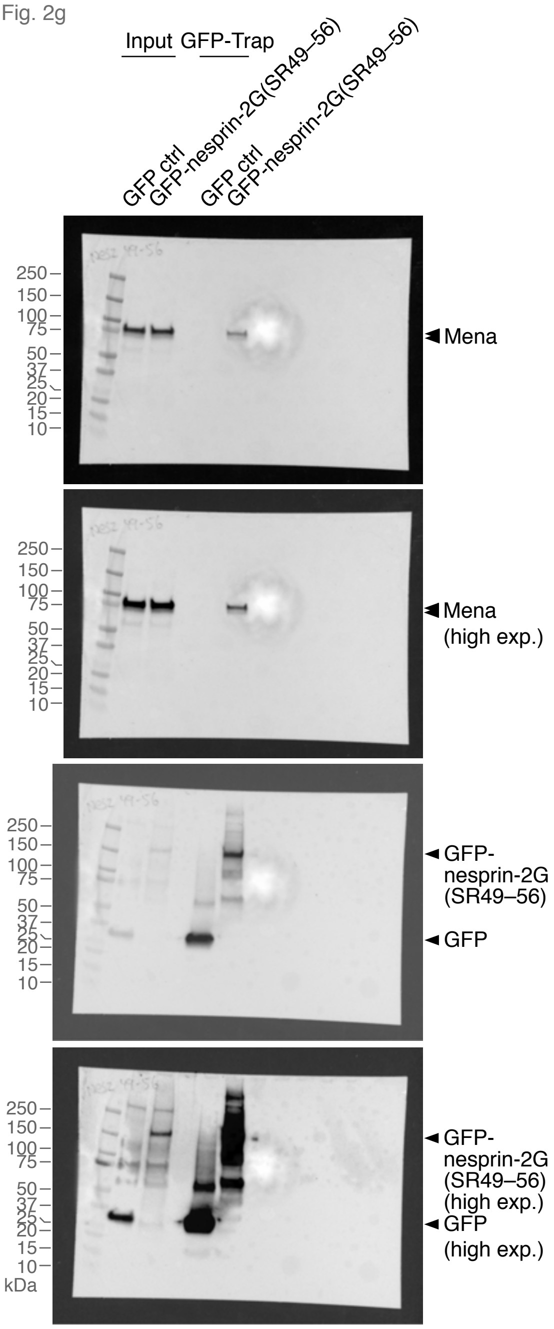

Fig. 2h

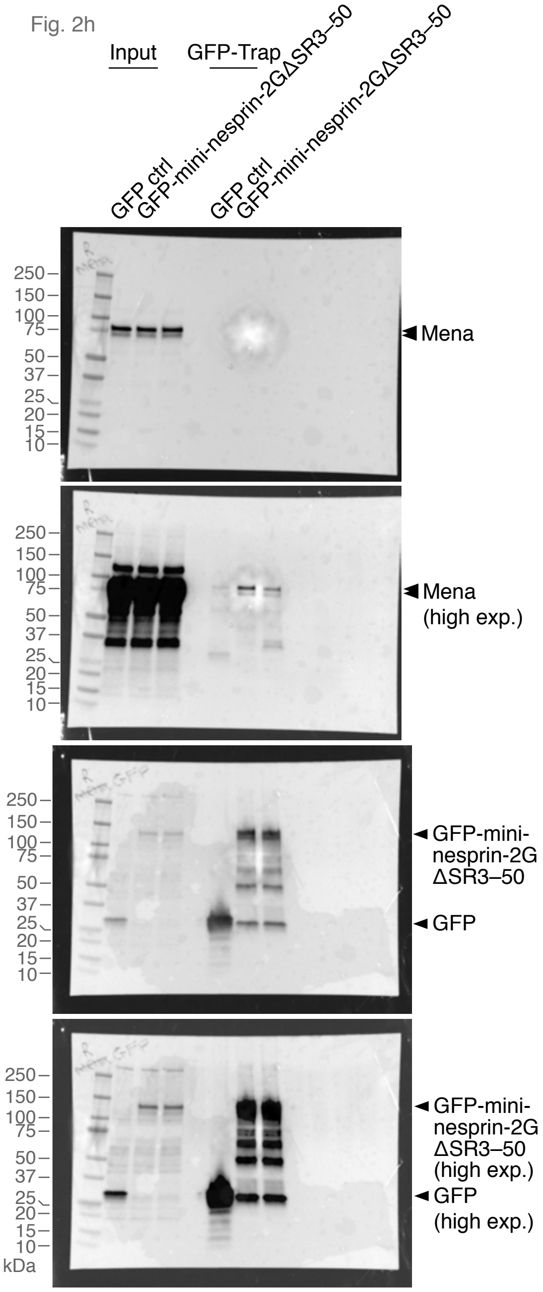

Source Data. Continued on next page.

Fig. 3a

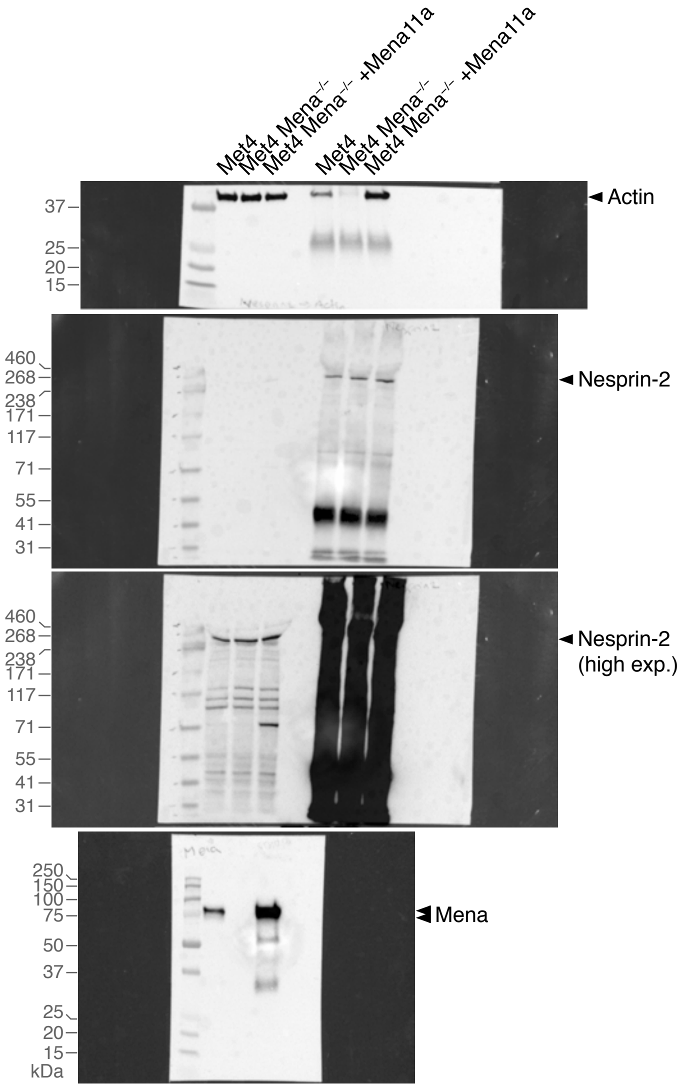

Fig. 3b

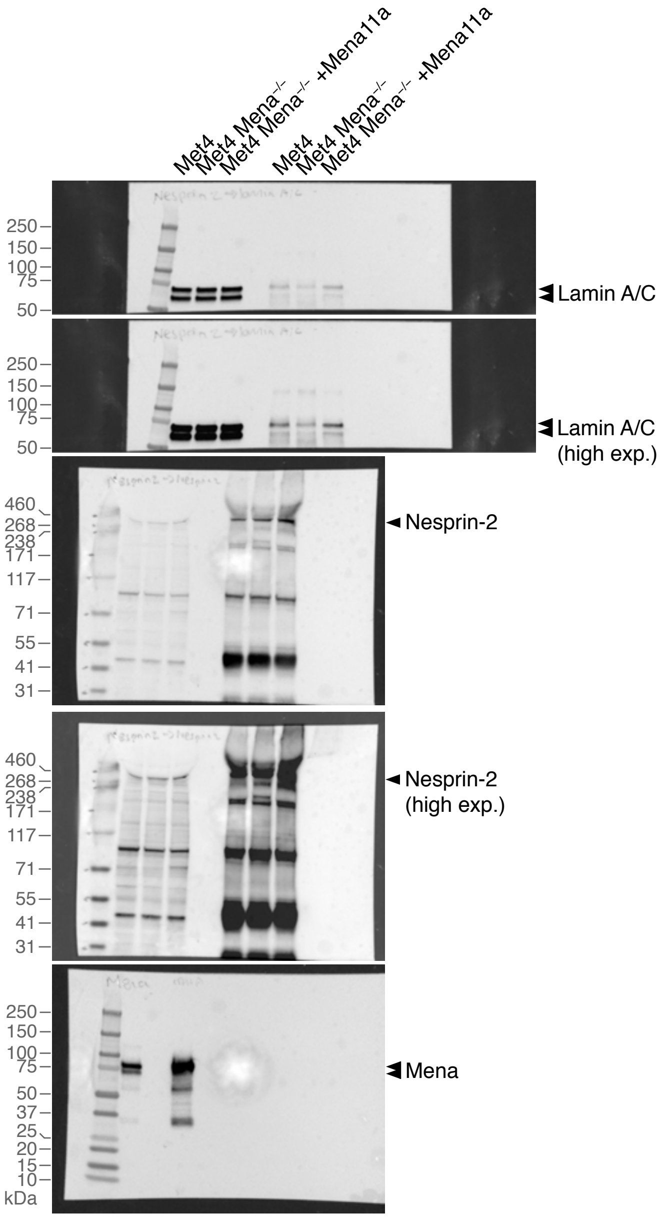

Fig. 4a

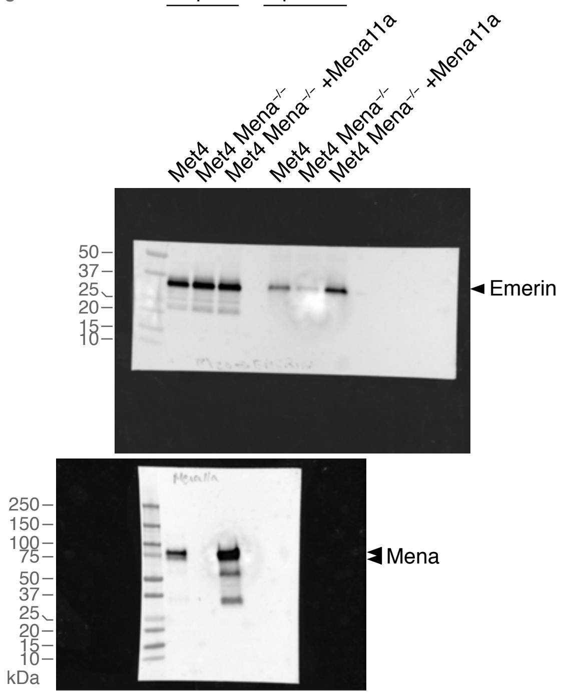

Source Data. Continued on next page.

Supplementary Fig. 1f

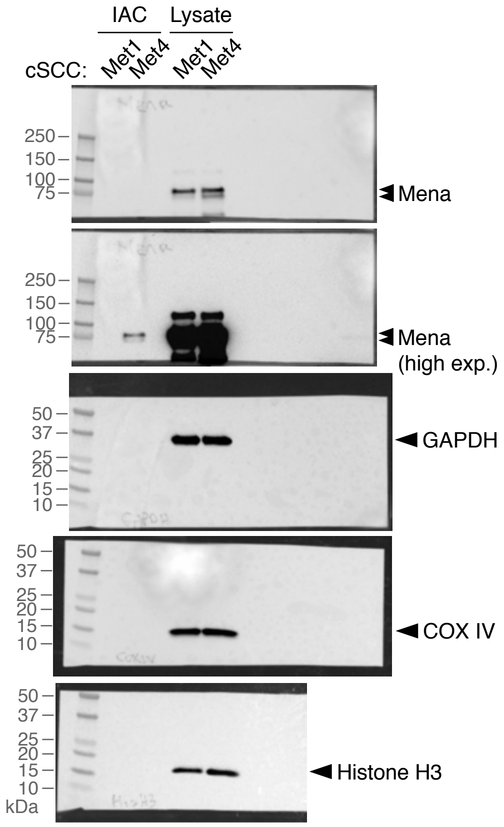

Supplementary Fig. 2a

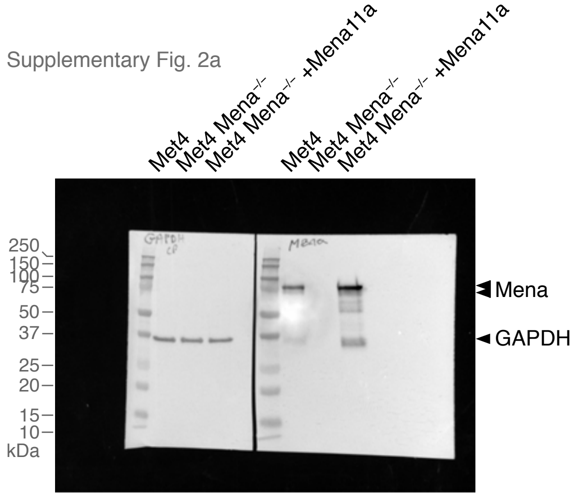

Supplementary Fig. 3a

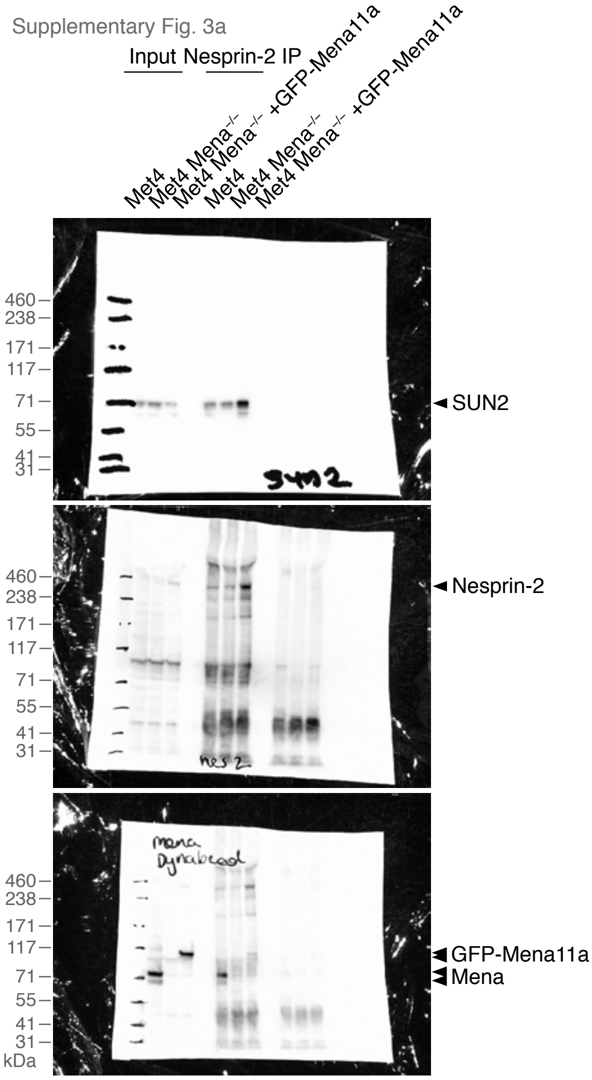

Supplementary Fig. 3c

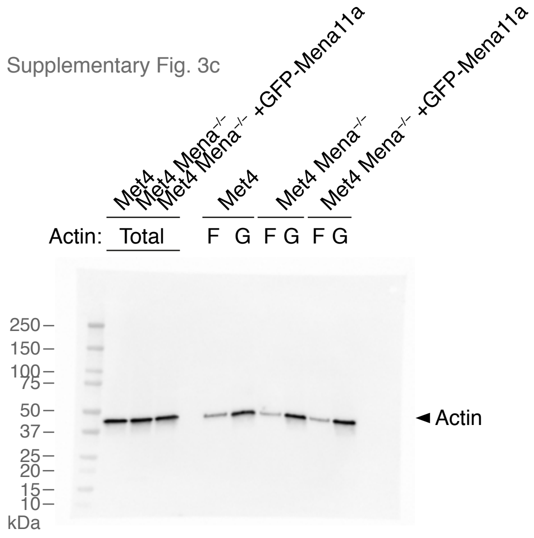

Source Data. Continued on next page.

Supplementary Fig. 4a

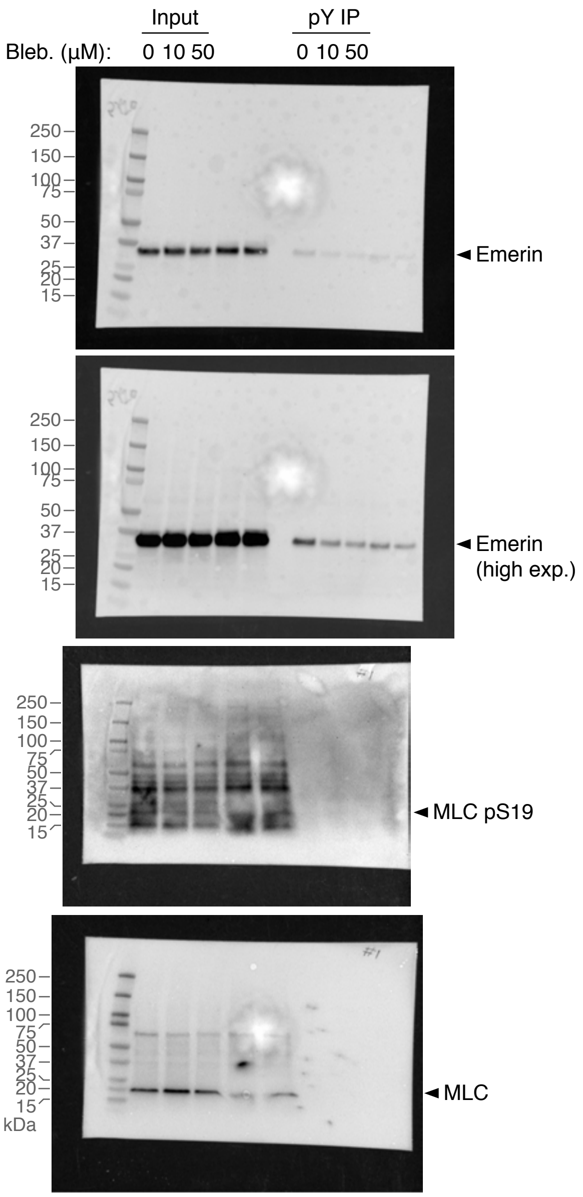

Supplementary Fig. 4b

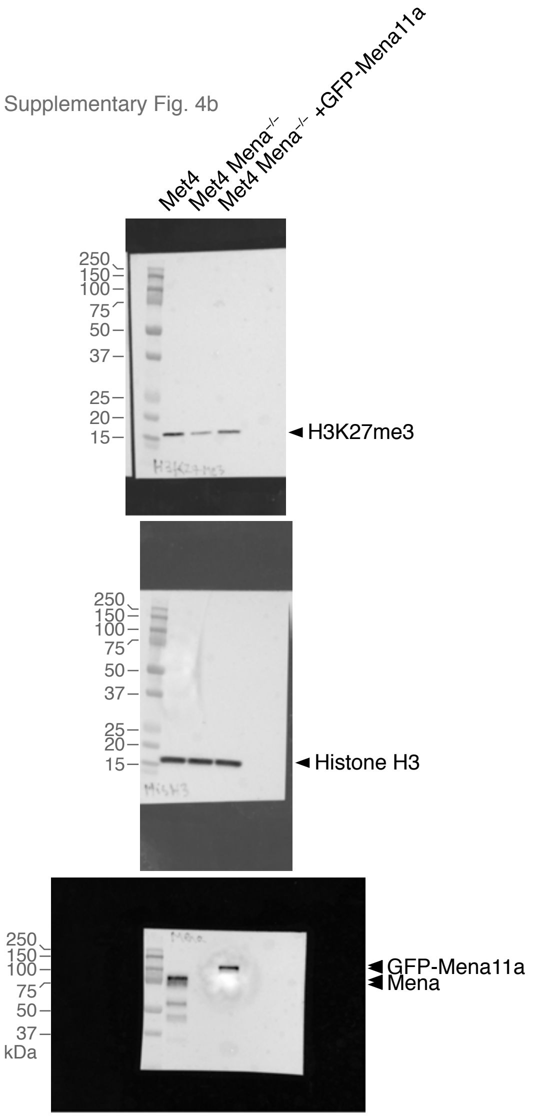

Source Data. Original blots. Arrowheads indicate expected band positions for each protein probed.
